# Multimodal fusion of pathology and radiology foundation models for WHO 2021 glioma subtyping

**DOI:** 10.1038/s41698-026-01366-5

**Published:** 2026-03-12

**Authors:** Camillo Saueressig, Daniel Scholz, Philipp Raffler, Claire Delbridge, Benedikt Wiestler, Peter Schüffler

**Affiliations:** 1 AI for Image-Guided Diagnosis and Therapy, Technical University of Munich, Munich, Germany; 2 Institute for Artificial Intelligence and Informatics in Medicine, Technical University of Munich, Munich, Germany; 3 Department of Neuroradiology, TUM University Hospital, Munich, Germany; 4 Institute of Pathology, Technical University of Munich, Munich, Germany; 5 Munich Center for Machine Learning, Munich, Germany; 6 Computational Pathology, Technical University of Munich, Munich, Germany

**Keywords:** Biomarkers, Cancer, Computational biology and bioinformatics, Medical research, Oncology

## Abstract

Molecular subtyping of gliomas is a common clinical task, yet challenging to perform on histology or radiology images alone. To address this challenge, we developed a multimodal classification framework that integrates histopathology and magnetic resonance imaging (MRI) using foundation models as unimodal experts, and evaluated three modality fusion strategies. Models are trained on two unpaired datasets of 772 histopathology cases and 959 multiparametric MRI scans, and tested on 171 unseen patient-matched cases. Multimodal models consistently outperform their unimodal counterparts, with a mixture-of-experts architecture achieving the strongest performance (AUC = 0.98 validation; AUC = 0.94 independent test set). Notably, we show that high-performing multimodal classifiers can be trained even without paired multimodal data, and in purely unimodal settings, these models match unimodal baselines. Finally, a detailed analysis of the learned multimodal representations reveals that the model identifies distinct visual biomarkers associated with glioma molecular subtypes, providing interpretable insight into its decision-making process.

## Introduction

Adult-type diffuse gliomas are the most common malignant primary brain tumors and account for an estimated annual mortality of 4.5 per 100,000 individuals^1,2^. According to the WHO 2021 classification guidelines, they comprise three genetic subtypes: IDH-wildtype glioblastomas, IDH-mutant 1p/19q-non-codeleted astrocytomas, and IDH-mutant 1p/19q-codeleted oligodendrogliomas^3^. Although these subtypes often present with similar symptoms and radiographic features, they differ markedly in prognosis and therapeutic management, underscoring the importance of accurate classification^1^. The gold standard for subtype determination is genetic testing, which can be time-consuming, expensive, requires extra tissue, and is not ubiquitously available^3^. As such, the ability to determine subtype from routinely collected clinical diagnostics such as multiparametric magnetic resonance imaging (mpMRI) and whole slide histology images (WSIs) could lead to faster diagnosis and treatment times.

Foundation models (FMs) are large-scale deep learning architectures trained on vast collections of unlabeled data, typically using self-supervised or unsupervised learning strategies^4^. Owing to this extensive pretraining, they serve as powerful and transferable feature extractors across a wide range of downstream tasks. Across clinical domains—including radiology, histopathology, and clinical reports—FMs have consistently enabled the development of high-performing classifiers for routine diagnostic and prognostic questions^4–7^. Recent work demonstrates that this holds true for glioma detection and molecular subtyping using both MRIs and WSIs^8–10^.

Building on these findings, we hypothesize that the complementary, modality-specific strengths of FMs can be combined to yield even more accurate and robust models for glioma classification. To this end, we use pretrained MRI and histology FMs as fixed feature embedders and train three multimodal classifiers employing distinct fusion strategies to predict WHO 2021 glioma subtypes. Conceptually, this approach parallels the workflow of a multidisciplinary tumor board, in which experts such as radiologists and pathologists contribute independent interpretations that are then integrated to guide clinical decision-making.

We then use these multimodal classifiers to address several challenges that are critical for both clinical adoption and methodological advancement. First, we demonstrate that combining pretrained MRI and histology foundation models enables a state-of-the-art multimodal classifier for WHO 2021 glioma subtyping, outperforming unimodal baselines as well as previous multimodal approaches^11–13^. Second, because patient-matched MRI-WSI datasets are rarely available at scale, we show that effective multimodal training is possible using entirely unpaired datasets, substantially lowering the data requirements for real-world deployment. Third, we evaluate model behavior when only a single modality is available at inference time and find that multimodal training produces classifiers that remain robust in both multimodal and unimodal settings. Finally, to ensure transparency and biological plausibility, we validate the learned radiographic and histologic biomarkers via attention maps and SHapley Additive exPlanations (SHAP^14^).

## Results

### Data composition

Ground truth labels were determined by genetic testing of biopsy or tumor resection, and are assigned according to the 2021 WHO classification as follows^3^: glioblastoma (IDH wildtype), astrocytoma (IDH mutant, 1p/19q intact), oligodendroglioma (IDH mutant, 1p/19q codeleted). Data characteristics are described in Table 1 and processing in Section “Data”.

**Table 1:** Dataset characteristics

	EBRAINS	UCSF	EGD	TCGA
Modality	WSI	MRI	MRI	Both
Glioblastoma	497	398	249	86
Astrocytoma	132	84	62	55
Oligodendroglioma	157	15	58	30

TCGA is the only dataset with patient-matched MRI and WSI.

### Multimodal models outperform unimodal equivalents

**Fig. 1:**
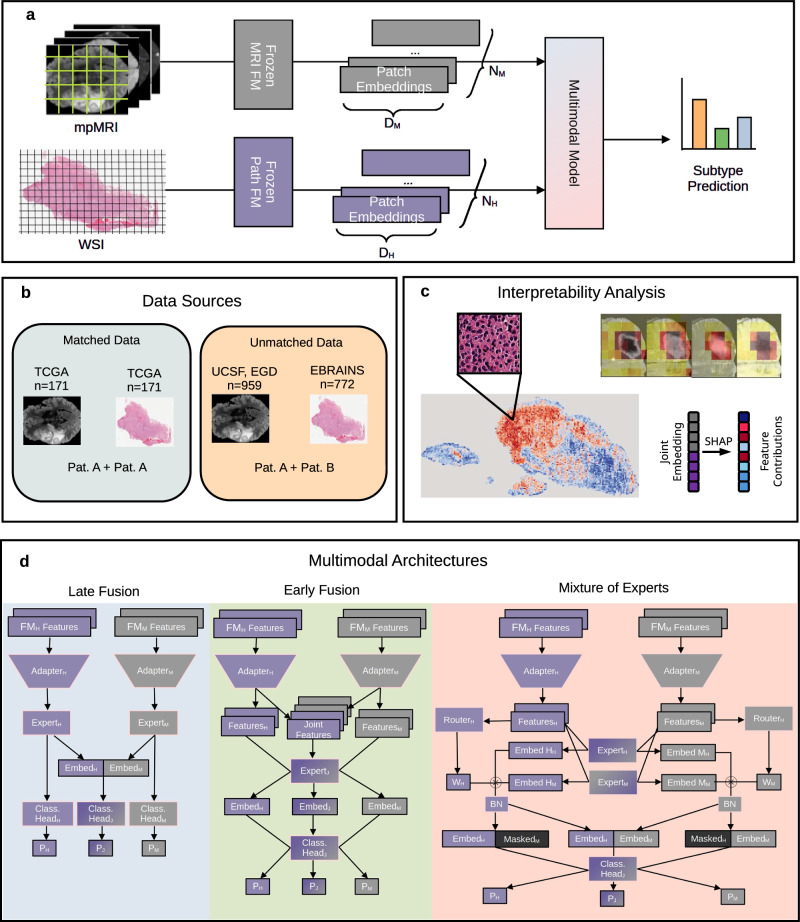
Study overview. **a** Both modalities are embedded by frozen foundation models to generate a token for each image patch. A multimodal model is trained to predict glioma subtype. **b** Patient-matched data is sourced from TCGA. Unmatched data from UCSF-PDGM, EGD, and EBRAINS. **c** Interpretability Analysis was performed via attention visualization and SHAP values. **d** The fusion architectures employed.

**Fig. 2:**
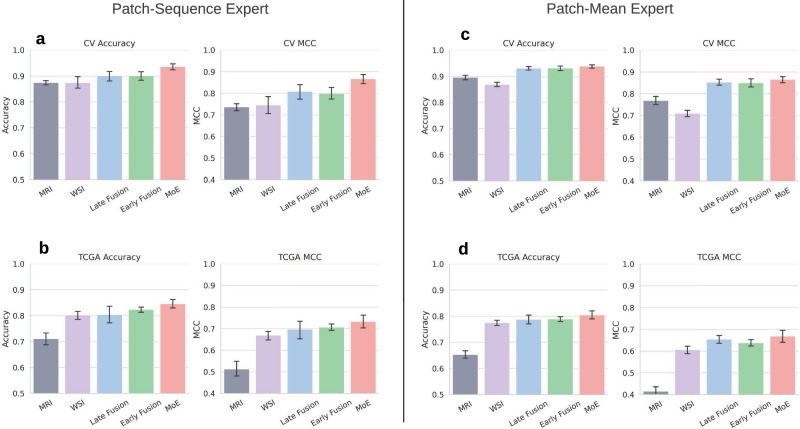
Accuracy and Matthew’s Correlation Coefficient (MCC) achieved during cross validation (CV) and on TCGA. Left **a**, **b**: Modality expert is a MambaMIL network. Right **c**, **d**: Modality expert is a linear layer. Multimodal models outperform unimodal models across all settings. Mixture of Experts (MoE) shows the strongest performance relative to other multimodal approaches, especially with patch-sequence expert. Patch-sequence expert is superior to patch-mean expert. Error bars represent standard deviation.

We train three multimodal architectures employing late fusion (MM-LF), early fusion (MM-EF), and a mixture of experts (MM-MoE) strategies (Fig. 1c). Each architecture is trained in two variants, employing either a lightweight linear expert (patch-mean) or a deep Mamba-based expert (patch-sequence)^15^. We compare their performance to unimodal networks in Fig. 2. Complete results across multiple metrics are given in Table S1. The multimodal networks consistently outperform the unimodal (UM-MRI, UM-WSI) networks, with the MM-MoE architecture showing the highest performance gain (MCC MM-LF: 0.70 ± 0.07, MM-EF: 0.71 ± 0.02, MM-MoE:0.73 ± 0.05). While UM-WSI and UM-MRI achieve comparable performance during cross-validation, UM-WSI (MCC 0.67 ± 0.04) greatly outperforms the radiology model (MCC 0.51 ± 0.06) on the TCGA (The Cancer Genome Atlas) test set. This finding suggests that pathology FM features generalize better than MRI FM features, which aligns with clinical experience, where histology is used as the dominant modality if genetic sequencing is not available.

We also characterize model performance stratified by subtype, with a specific focus on cases where the WHO 2021 classification differs from the initial histomorphological assessment, i.e., those cases where the histomorphology is discordant with the final, molecular diagnosis. These results are presented in Table 2. Of the 171 TCGA cases, 52 (30%) received an updated diagnosis according to WHO 2021 criteria: 25 of these were initially classified as oligoastrocytomas, a subtype which no longer exists. In general, all models perform better on cases whose initial diagnosis is consistent with the WHO 2021 diagnosis (70% of cases), likely because the genetic and morphological features overlap. Moreover, all models at least match the overall 70% accuracy of the initial pathologist diagnosis (when WHO 2021 labels are considered ground truth), providing strong unimodal baselines.

**Table 2:** Model accuracy reported on holdout TCGA data

Hist. Diag.	WHO21 Diag.	Count	UM-MRI	UM-WSI	MoE-MRI	MoE-WSI	MoE-MM
gbm	gbm	78	0.87 ± 0.07	0.87 ± 0.08	0.88 ± 0.07	0.88 ± 0.07	**0.94 ± 0.04**
astro	astro	21	0.44 ± 0.04	**0.75 ± 0.11**	0.40 ± 0.06	0.61 ± 0.12	0.73 ± 0.09
oligo	oligo	20	0.46 ± 0.08	**0.72 ± 0.06**	0.45 ± 0.05	0.69 ± 0.03	0.69 ± 0.05
**consistent**	**consistent**	**119**	0.73 ± 0.07	0.83 ± 0.08	0.72 ± 0.06	0.80 ± 0.07	**0.86 ± 0.05**
oligo	astro	13	**0.71 ± 0.06**	0.58 ± 0.20	0.69 ± 0.04	0.61 ± 0.18	**0.71 ± 0.07**
astro	oligo	9	0.80 ± 0.1	0.86 ± 0.05	0.86 ± 0.11	0.90 ± 0.06	**0.97 ± 0.05**
oligo	gbm	5	0.84 ± 0.08	0.76 ± 0.21	0.80 ± 0.00	**0.86 ± 0.13**	0.84 ± 0.16
OA	astro	21	0.53 ± 0.06	**0.88 ± 0.08**	0.47 ± 0.07	0.85 ± 0.10	0.86 ± 0.06
OA	gbm	3	**0.83 ± 0.18**	0.43 ± 0.22	0.70 ± 0.11	0.60 ± 0.14	0.50 ± 0.18
OA	oligo	1	**1.00 ± 0.00**	0.10 ± 0.32	**1.00 ± 0.00**	0.20 ± 0.42	0.9 ± 0.32
gbm	astro	0	–	–	–	–	–
gbm	oligo	0	–	–	–	–	–
astro	gbm	0	–	–	–	–	–
**updated**	**updated**	**52**	0.68 ± 0.06	0.75 ± 0.11	0.65 ± 0.05	0.77 ± 0.10	**0.82 ± 0.07**

The cohort was divided into cases where the initial histomorphological diagnosis was **consistent** with 2021 WHO criteria and those that had their diagnosis **updated** based on genomic integration. Consistent cases (70%) are those where the initial histological (pathologist visual inspection) diagnosis was correct, while the remaining 30% were initially misclassified by pathologists. *gbm* glioblastoma, *astro* astrocytoma, *oligo* oligodendroglioma, *OA* oligoastrocytoma. Our multimodal model (MoE-MM) achieves the highest overall accuracy on both consistent and updated cases. The unimodal histology model (UM-WSI) achieves the highest accuracy for histology-genomic-consistent astrocytomas and oligodendrogliomas. Multimodal improvement over unimodal is most pronounced in updated cases. The accuracy of the best model(s) for each row is displayed in bold. Accuracy reported as mean ± std over 10 folds.

Nonetheless, the best-performing MoE-MM architecture outperformed unimodal variants on both consistent and updated cases, reaching 85% overall accuracy. The greatest improvement over unimodal models is observed in distinguishing updated astrocytomas and oligodendrogliomas. Astrocytomas and oligodendrogliomas are commonly grouped together into low-grade, IDH mutant gliomas and are difficult to distinguish in the absence of typical morphology, typically requiring genetic differentiation. We hypothesize that the addition of a second modality facilitated classification by providing additional context and enabling the learning of cross-modal interactions. Similarly, the second modality synergistically improved glioblastoma classification beyond the levels achieved by either modality alone (Figs. 2 and S1). Another common subtype misclassification, glioblastoma and high-grade astrocytoma (WHO grade 4), is not represented in the data.

### Multimodal learning without patient-matched data

Patient-matched multimodal data is often difficult to acquire in large quantities, impeding the training of robust models. By contrast, unimodal datasets with shared labels are often readily available, but ignored for multimodal tasks. Glioma subtyping is no exception to this rule, with previous work employing specially curated datasets with fewer than 400 patients^16^. By leveraging three unimodal datasets, we can train on 1731 unique patients, with random sampling yielding tens of thousands of multimodal permutations during training.

**Fig. 3:**
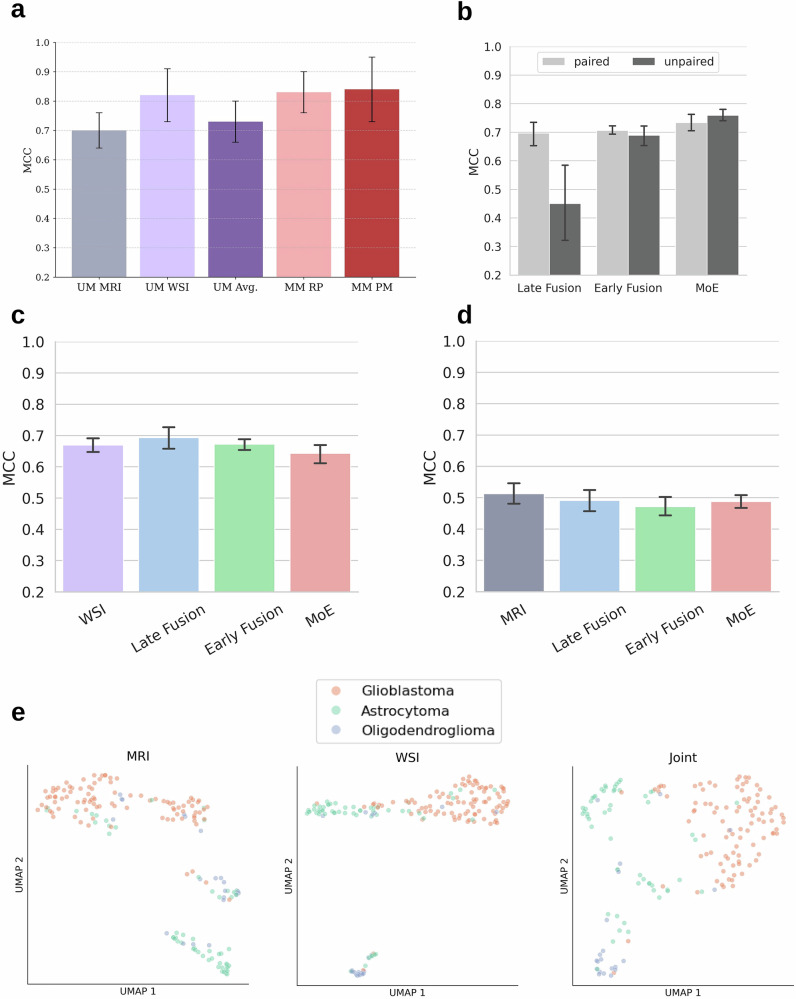
Comparison of data sampling strategies and per-modality performance. **a** Cross-validation results on TCGA. MM-RP (random pairing) is nearly as good as MM-PM (patient-matched) training, and superior to UM-Avg (average MRI+WSI prediction). In-domain UM-WSI is nearly as good as multimodal fusion. **b** TCGA (holdout test) performance of models trained with random pairing (paired) vs. trained alternately on single modalities (unpaired). Unpaired training is equivalent to random pairing, except for late fusion architecture. **c** Performance on TCGA WSIs by architecture. There is no significant difference in performance for unimodal model (UM-WSI) and multimodal models. **d** Same for MRI. **e** Final layer embeddings of Mixture of Experts (MoE) model when different modalities are available. Left: MRI-only features. Middle: WSI-only features. Right: Joint MRI-WSI features. Embeddings are projected to two dimensions using UMAP and colored according to true label. The joint embedding projection produces better-separated clusters than either modality alone. Error bars represent standard deviation.

We first show, using the paired samples available (TCGA), that random label-pairing achieves performance equivalent to fully patient-matched training and is superior to naive ensembling of unimodal models (Fig. 3a). Subsequently, we further explore unmatched training on the full dataset (Fig. 3b). We define label-paired training as randomly sampling an MRI and a histology case with the same label at each training epoch, and unpaired training as alternating between batches of MRIs and WSIs. Surprisingly, we find that completely unpaired training performs just as well as random label-pairing, with the exception of MM-LF (two-tailed Mann–Whitney U test for difference in Matthews’s Correlation Coefficient (MCC), *n* = 10. MM-LF: *p* = 0.04; MM-EF: *p* = 0.45; MM-MoE: *p* = 0.17). Unlike MM-EF and MM-MoE, the joint output head of MM-LF cannot be trained in an unpaired setting, so we instead compare to naive ensembling. In order to exclude pairing-induced regularization as the source of increased performance, we train a MoE-MM model on label-agnostic random pairing and observe the performance degrade to the level of UM-WSI (TCGA MCC ± std = 0.67 ± 0.07).

### Multimodal models retain unimodal capability

While clinics routinely acquire both MRI and WSI data from glioma patients, there are cases where only one of the two modalities is present. For example, a surgeon may wish to obtain an estimate of tumor subtype preoperatively in order to better plan a surgery. In this case, a classification model must be able to handle a single modality. We find that, for both histology and radiology, the multimodal models match the performance of the equivalent unimodal models when evaluated with only one modality present (Fig. 3c, d; two-tailed Mann–Whitney U test for difference in MCC, *n* = 10. UM-LF vs. UM-WSI: *p* = 0.27; UM-EF vs. UM-WSI: *p* = 1; UM-MoE vs. UM-WSI: *p* = 0.25; UM-LF vs. UM-MRI: *p* = 0.62; UM-EF vs. UM-MRI: *p* = 0.08; UM-MoE vs. UM-MRI: *p* = 0.38). This finding implies that one generalist model can be used for any number of modalities, rather than needing an individual model for each one.

To further characterize the MoE model, we visualized the final layer features from MM-MoE in the multimodal and both unimodal scenarios using UMAP (Fig. 3e). Visually, the multimodal embedding creates more distinct clusters, in particular, disentangling glioblastomas and astrocytomas. Quantitatively, we performed k-means clustering on the embeddings and computed the Fowlkes– Mallow Index (FMI) between the clustering-predicted class distribution and the true class distribution. The FMI was 0.79 for the joint embedding and 0.73 and 0.55 for histology and radiology, respectively, further emphasizing the superior discrimination ability of the multimodal model.

### Multimodal models exhibit more diffuse attention

**Fig. 4:**
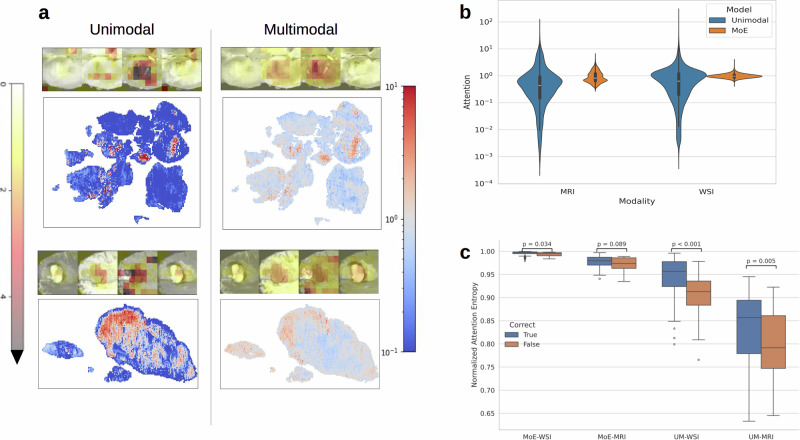
Attention analysis for MM-MoE vs. UM-WSI and UM-MRI. Attention is scaled such that a value of 1 is equal attention to all slides. **a** Examples of attention distribution. WSI visualizations are log-scaled. MRI visualizations are linearly scaled. Unimodal and MoE-MM attend to similar regions, but unimodal attention is more polarized. **b** Unimodal attention is highly specific, with individual patches receiving up to 10^5^ more weight than others. Multimodal attention is more balanced, with the median attention value being close to 1. **c** Boxplot of attention entropy grouped by correct prediction and model. Images with higher entropy are more likely to be correctly classified. WSIs have a higher entropy than MRIs.

Next, we examined whether model attention could provide evidence for subtype-associated biomarkers. We visualized attention maps for both modalities and examined attention patterns from the MoE model and the respective unimodal models (Fig. 4). Both models primarily attended to tumor-rich areas. MM-MoE exhibited more diffuse attentionacross the entire tissue sample/scan compared to the unimodal models. Whereas unimodal models frequently hyper-focused on isolated regions or patches, the MoE identified the same key regions but simultaneously integrated the surrounding context. For example, MoE attention is distributed across all four MRI sequences, whereas the unimodal MRI model concentrates predominantly on T1c. Despite these differences, the two approaches produced similar performance in the unimodal setting (Fig. 3c, d).

Building on this finding, we identified an association between attention diffuseness and performance by quantifying the attention entropy of each image (Fig. 4c). Counterintuitively, images with higher entropy (i.e., more diffuse attention) were more likely to be predicted correctly. However, this difference is driven largely by differences between subtypes, with the most performant class (glioblastomas) also having the highest median attention entropy S5. One possible explanation for this unexpected attention behavior is that the model scans the image for positive evidence of low-grade glioma biomarkers, which would then receive a high attention weight.

### Relative feature importance

In order to further elucidate the contribution of certain histologic or radiologic features to model predictions, we performed a SHAP value analysis on MoE embeddings extracted from the model prior to the classification head. SHAP is a game-theoretic approach that allocates credit to each input feature based on how much its presence or absence affects the model prediction^14^. We identified certain features that strongly contribute to each individual class^5^. For example, position 19 (F19) and position 5 (F5) of the embedding contribute the most to glioblastoma prediction. Moreover, by grouping contributions by genetic status rather than the tumor subtype, we were able to identify features that contribute to IDH mutant and 1p19q codeletion predictions. A common trend across both analyzes is that histology features contribute more to the final prediction than radiology features. Of the two mutations, radiology is more helpful for determining IDH status.

### High SHAP value features have distinct morphological correlates

**Fig. 5:**
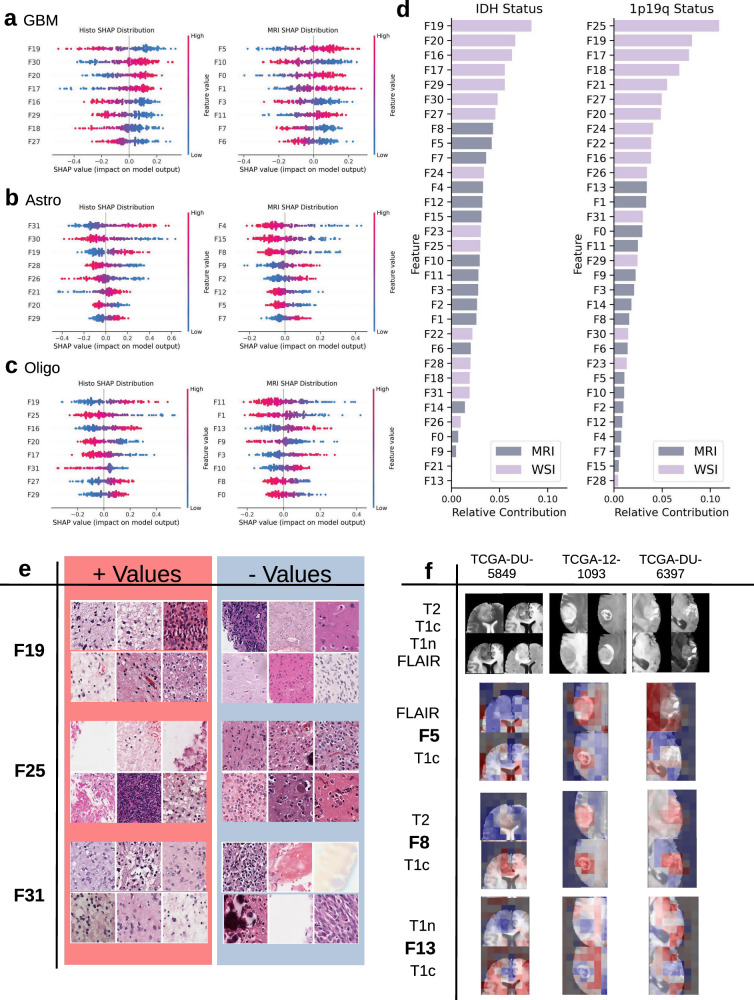
SHAP value analysis. **a**–**c** Contributions of the top 8 features for prediction of each class. Color indicates the numerical value of each feature, while position on the *x*-axis indicates a contribution towards or away from predicting that class. **d** Absolute relative contribution of each feature towards differentiating IDH status and 1p19q status. **e** Example patches for high and low values of three histology features. Higher values of F19 are associated with IDH-mutant histological features. F25 encodes oligodendroglioma morphology. F31 correlates with astrocytoma patches. **f** Example visualizations of selected MRI features. F5 highlights ventricles, F8 highlights core tumor, and F13 highlights white matter tracts.

Next, we visualized and reviewed image regions with highly positive or negative values of the most important features to determine whether features correspond to specific morphological features (Fig. 5f).

We first examined the most influential MRI-derived contributors: Features 5, 8, and 13 (F5, F8, F13). High values of F5 were found to correspond to cerebrospinal fluid (CSF), in particular, the ventricles in FLAIR and T1c sequences, while low values were found, e.g., in healthy white and grey matter. F8 showed a strong association with tumor tissue, especially regions of contrast enhancement on T1c. In contrast, healthy brain tissue was either not represented by F8 or, in T2, associated with negative F8 values. According to our SHAP analysis, F5 and F8 were the principal MRI features driving IDH-status prediction. While high F8 values (reflecting contrast enhancement and diffuse tumor growth) align with known radiographic hallmarks of high-grade, typically IDH-wildtype gliomas, the contribution of F5 is less intuitive. A plausible explanation is that the generally more aggressive growth behavior of IDH-wildtype diffuse gliomas leads to greater mass effect compared with IDH-mutant diffuse gliomas, which may in turn result in midline shift, obstruction of CSF pathways, and thereby ventricle enlargement.

In contrast, F13 contributed primarily to the prediction of 1p/19q codeletion status and did not influence IDH classification. F13 corresponded to normal-appearing white matter, particularly in T1n and T1c, the sequences with the highest anatomical fidelity. This suggests that the presence of preserved white matter may help distinguish oligodendrogliomas from astrocytomas.

The most prominent histology feature was F19, which plays a leading role in subtype prediction for all three classes. Analysis of patches with high F19 values revealed both astrocytic and oligodendrogliomic features, with a focus on prominent nuclei and fibrous, spongy background. By contrast, low F19 correlated with both healthy tissue and polymorphic hypercellularity, as can be seen in glioblastomas. Taken together, these observations indicate an IDH-mutation detector function for F19, which is confirmed by the mutation-level analysis (Fig. 5). We hypothesize that F19 also contributes to 1p19q codeletion detection since IDH mutation is a prerequisite for the second mutation. Along with F19, the most meaningful feature for 1p19q codeletion detection is F25. Indeed, low values of F25 are found on patches with typical oligodendroglioma features such as homogeneous, round nuclei surrounded by perinuclear halos. High values were not associated with a particular pattern, indicating that F25 may encode the concept of “oligodendroglioma-ness”, ranging from no similarity to typical morphology. F31 is the leading feature for astrocytoma classification. We find an emphasis on loose, microcystic background, moderate nuclear polymorphism, as well as clearly delineated perikarya. Low F31 is observed in a variety of patches, including background, out of focus, and some oligodendroglioma patches. Accordingly, we interpret this feature as primarily astrocytoma-specific, with some ability to distinguish between oligodendrogliomas.

Interestingly, none of the top features encode positive evidence for glioblastomas, e.g., neovascularisation or pseudopalisading necrosis. As postulated in Section “multimodal models exhibit more diffuse attention”, the model seems to treat glioblastoma as the default subtype, and primarily looks for evidence to refute it. This interpretation is supported by the magnitude of the SHAP values, as SHAP values contributing towards predicting glioblastoma are much smaller than those pulling away from it (Fig. 5a).

## Discussion

In this study, we developed a multimodal glioma classification model trained exclusively on unmatched MRI and WSI data. Our results demonstrate that the multimodal model outperforms unimodal approaches by 9% (WSI) and 43% (MRI) when both modalities are available, while achieving comparable accuracy to unimodal models when only a single modality is present. This enables the model to function as a single, unified classifier, regardless of how many modalities are available. Moreover, we establish random-pairing or unpaired training as feasible alternatives to patient-matched data, and identify multiple imaging biomarkers consistent with the literature.

Multimodal fusion for glioma classification has previously been explored by a select number of groups^17^. Nearly all previous studies have attempted this task in the context of the CBM Rad-Path challenge or subsets of the dataset, consisting of 221-388 patient-matched cases^16^. Unfortunately, we were unable to locate the CBM Rad-Path dataset for direct comparison. Typical approaches include CNN-based unimodal models with prediction-level fusion^11,12^, or post-CNN feature concatenation^18^. Mallya et al. describe a histology-guided MRI classification model, whose performance approaches a joint histology-radiology model^13^. These approaches achieved balanced accuracy (BA) scores between 0.777 and 0.889 on fivefold validation, and 0.654 and 0.750 on a small test set of 40 patients. Albuquerque et al. explore the utility of different MRI modalities and fusion strategies on the TCGA dataset, achieving an overall accuracy of 0.83 in fivefold validation^19^. By contrast, we report a BA of 0.91 on fivefold validation and 0.80 on an out-of-domain test set consisting of 171 patients. Moreover, we achieve this state-of-the-art performance by leveraging existing foundation models and using lightweight downstream classifiers of less than a million parameters.

A related body of work focuses on glioma survival prediction^20^. These studies often train and validate their models entirely within TCGA, as it is one of the few datasets with a meaningful number of matched patients. One of the earliest works is a Cox proportional hazards regression model trained on handcrafted radiology and pathology features to predict overall survival^21^. Later methods expanded this approach to intelligently fuse histology and radiology imaging with clinical and genetic data, and to account for missing modalities^22,23^. More recent work has employed pretrained histology embedders that encompass the whole WSI, rather than just a selected region, and focus on cross-modal attention mechanisms^24,25^.

Unlike previous work, our model is trained entirely on non-patient-matched data. Our results suggest that both unpaired and randomly label-paired approaches are viable alternatives to patient-matched data. This strategy benefits from multiple advantages. Firstly, we are able to leverage large, publicly available datasets for multimodal classification training which were previously ignored. Subsequently, we can report results on the withheld paired cases as an unseen external test set, which would otherwise be infeasible with only one patient-matched dataset. Moreover, randomly paired training functions as a form of data augmentation. In a real-world setting, a given MRI might be associated with multiple different histology slides, depending on the site of biopsy, in particular for highly heterogeneous gliomas^26,27^. Our approach allows us to simulate this heterogeneity by pairing each MRI with both typical and atypical histology and vice versa, leading to more robust subtype recognition.

Another key improvement in our method is the use of foundation models (FMs) as feature extractors for both histology and radiology images. FMs are trained on massive datasets (e.g., billions of image patches), enabling them to produce substantially more expressive embeddings than conventional task-specific encoders. Additionally, their self-supervised pretraining paradigm forces FMs to produce consistent embeddings across different views. These embeddings have been shown to generalize well to both diverse downstream domains and downstream tasks, often outperforming task-specific models^28,29^. Recent work has shown that while pathology foundation models do encode scanner information, they do not suffer a drop in performance on account of scanner variation^30^. Taken together, these properties suggest that explicit modeling of domain shift is less critical in this setting, which is further supported by the strong performance of our method on both in-domain and out-of-domain data, even in the absence of stain normalization or data augmentation (Figs. 2 and S2).

Our interpretability analysis enabled us to validate the model’s decision-making against established biomarkers and to gain insight into which diagnostic cues our model learned to encode. We observed that individual features within the multimodal embedding captured either subtype-specific features, such as contrast enhancement on T1c MRI or perinuclear halos in histology, or more general image attributes, including the presence of CSF or overall cell morphology. Notably, we identified one feature (F19) that appeared strongly associated with IDH mutation status and another (F25) that seemed to estimate 1p19q codeletion by quantifying how closely a histology patch resembled classic oligodendroglioma morphology.

Several expected morphological correlates, however, were not utilized by the model. For example, we did not identify any feature directly corresponding to midline shift in MRI, nor any representation of cellularity in WSIs— both commonly used clinical indicators. Additionally, histologic hallmarks of glioblastoma were underrepresented, likely because glioblastoma constitutes the majority class and thus becomes the model’s default representation. Future biomarker-discovery efforts may benefit from incorporating healthy tissue as an additional class, which could help the model learn more specific and discriminative features for glioblastoma.

Clinically, the gold standard for molecular glioma subtyping is based on molecular assessment of the tumor tissue, but these results only become available days to weeks after surgery. Ideally, the subtype can be determined prior to surgery so it can inform not only post-surgery treatment, but also the surgeon’s strategy and the extent of resection^31^. Unfortunately, subtype prediction from pre-operative MRI alone is often imprecise, as our results confirm^32^ (Fig. 2). In this study, we take an intermediate approach of combining pre-operative MRI with formalin-fixed paraffin-embedd (FFPE) histology slides. These slides typically become available hours to days after surgery, providing faster, cheaper results than molecular assessment. A natural extension to our approach is to replace the post-operative FFPE tissue with tissue available prior to or during surgery, such as biopsies, cryosection slices, or simulated Raman histology^33,34^. Our approach remains performant even at fewer than 100 patches, suggesting that deployment on biopsy data could be possible (Fig. S2). Given scarce data availability, model development is difficult, but these extensions would continue to enable the high accuracy achieved by our approach, while being available during surgery.

Data-related constraints also limit the generalizability of our results. Although we include the entire TCGA paired cohort as an unseen test set, the lack of publicly available paired MRI-pathology data impedes the clinical validation of our results. Given the unpaired nature of our data, meaningful single-center analysis of the training datasets to characterize robustness is also difficult. We attempt to address this limitation via acquisition-site stratification (Fig. S2), but recognize that further independent validation is necessary before clinical adoption can be considered. Another limitation of our approach is that it incorporates only two data types; integrating clinical reports, demographic variables, and other patient-level information may improve predictive robustness and clinical relevance. In addition, the MRI foundation model processes only 2D slices, which prevents it from capturing the full 3D spatial context and tumor localization, potentially limiting its representational power. We attempt to address this limitation via multi-scale concatenation (2.5D), but see no improvement over one 2D slice (Fig. S2). Finally, our evaluation focuses only on subtype prediction. Future extensions should explore further clinically relevant outcomes such as survival and treatment response, which are likely to also benefit from integrated multimodal modeling.

Other exciting extensions include more comprehensive validation and smarter multimodal integration. Although multimodal fusion clearly improves subtype classification, our interpretability analyzes examine MRI and histology separately, leaving open the opportunity to explore explicit cross-modal interactions and reveal how complementary signals jointly inform predictions. Differences in fusion performance across architectures suggest room to design more interaction-aware multimodal models that better capture shared biological structure. We also found no clear benefit of multimodal training for unimodal MRI prediction, suggesting that our current fusion strategies may not effectively transfer histological information into MRI representations. Developing a more explicitly histology-informed MRI encoder remains a vital next step in bridging the gap between non-invasive imaging and cellular-level pathology.

## Methods

### Data

MRI data were sourced from UCSF-PDGM (University of California San Francisco, Preoperative Diffuse Glioma MRI *n* = 497^35^), EGD (Erasmus Glioma Dataset, *n* = 462^36^), and TCGA (*n* = 171^37,38^). Only cases with genetic testing sufficient to establish a WHO 2021 diagnosis and T1, T1c, T2, and FLAIR sequences were included. Histopathology data were acquired from EBRAINS (*n* = 772 cases^39^) and TCGA (*n* = 171). EBRAINS, UCSF-PDGM, and EGD labels were converted from WHO 2016 to WHO 2021 labels based on IDH and 1p/19q status. TCGA labels for both datasets were taken from de Mendonça et al.^40^. If a case consisted of multiple WSIs, all were included. A small number of WSIs with insufficient tissue area for segmentation and patching were excluded. All data used in this study were publicly sourced, and so ethics approval was not requested for this study.

### Data preprocessing

MRIs were processed as described in Scholz et al.^9^. All MRIs were resampled to 1 × 1 × 1mm isotropic resolution and rigidly registered to the SRI24 atlas^41^. The axial middle slice of the tumor was cropped to 96 × 96 around the center of mass of the tumor segmentation mask, and normalized inside the brain mask to a [0,1]-range.

WSIs were divided into bags of patches as described in Chen et al. (2024)^42^. Briefly, WSIs were segmented by binary thresholding to remove background and tessellated into 256 × 256 patches. We performed no stain normalization or data augmentations prior to FM encoding.

### Foundation model patch encoding

MRI slices were encoded using MM-DINOv2^9^. First, the MRI slices were resized to 98 × 98. Each of the four MRI sequences was converted to an RGB image by stacking each sequence three times and normalized to mean (0.485, 0.456, 0.406) and standard deviation (0.229, 0.224, 0.225), which corresponds to the original DINOv2 normalization. Inside MM-DINOv2, all MRI sequences were divided into patches of size 14 × 14. MM-DINOv2 yields 49 embeddings for each of the four MRI sequences used and one global embedding, each with 768 dimensions, yielding an MRI feature embedding of 197 × 768.

Histology patches were encoded using Prov-GigaPath^28^. Prov-Gigapath was chosen asstrong empirical performance on glioma classification has previously been observed^8^. For each WSI, all patches are resized to 224 × 224, Z-normalized, and transformed into feature embeddings. After embedding, each WSI is represented as a variable-length tensor of shape *N*_*H*_*xd*_*H*_, where *N*_*H*_ is the number of patches and *d*_*H*_ is the embedding size, 1536. Each image was encoded once using a frozen FM prior to training.

### Model architectures

Each model is composed primarily of an adapter and an expert network for each modality, followed by a joint classification head. The input to each model is a sequence of FM-extracted embeddings for each modality, as described in Section “foundation model patch encoding”. The adapter is a single linear layer that projects the embeddings to a common dimension *d*_*C*_. The classification head is a 2-layer multilayer perceptron (MLP) with a ReLU nonlinearity. We implemented a low-parameter expert, which condenses each modality to only the mean patch embedding (patch-mean), and a high-parameter expert, which operates on the entire sequence (patch-sequence). The patch-mean variant consists of a feature-wise mean, a ReLU nonlinearity, and a linear layer. As such, patch-mean models form a 4-layer MLP. Total parameter count is approximately 110k and 220k parameters, for unimodal and multimodal models, respectively. Patch-sequence experts consist of *n* Mamba blocks followed by attention pooling^15,43^. *n* = 12 for multimodal models and *n* = 24 for multimodal models to match parameter count as closely as possible between models (between 900k and 950k for all patch-sequence models).

Unimodal models (UM-MRI, UM-WSI) consist solely of one adapter, expert, and classification head as described above. MM-LF separately processes each modality, followed by concatenation and a joint classification head (Fig. 1). Two additional classification heads allow for unimodal classification. MM-EF instead fuses the modalities prior to the expert network, and consequently, only has one expert and classification head. Patch-sequence models accomplish feature fusion through embedding concatenation in the embedding dimension, resulting in one sequence of shape (*N*_*H*_ + *N*_*M*_) × *d*_*C*_. Patch-mean models instead fuse through mean-pooling the representations from each modality. MM-MoE is adapted from Xu et al.^44^. After feature adaptation, both modalities are processed by both expert networks, and a soft router dynamically weights the contribution of each expert. Unlike Xu and Jiang et al., we use one router per modality. To simplify the computation, the router weight is computed on the mean embedding, even for patch-sequence models. We extend the incomplete multimodal training strategy investigated by Xu and Jiang et al. by always training on all input permutations, i.e., for a given pair of inputs, we predict both unimodal outputs as well as the multimodal output. As in Xu and Jiang et al., a zero vector is used to replace masked modalities in the unimodal case.

A shared dimension *d*_*C*_ of 64 was empirically determined to offer the best performance. In order to simplify interpretation of individual features, *d*_*C*_ = 16 was instead chosen for MoE as performance was observed to be stable w.r.t embedding dimension.

### Model training

Data from EGD, UCSF, and EBRAINS were pooled and divided into fivefolds, which was used for training and cross-validation. Folds were constructed to contain representative proportions of each label and stratified by case. To account for variation in random sampling (see below), each fold was trained twice, for a total of 10 trained models. TCGA cases were reserved as an independent test set. MRI cases which were used for training of MM-DINOv2 were excluded from validation folds. Prov-GigaPath was not trained on either EBRAINS or TCGA, preventing potential data leakage.

Models were trained with a batch size of 32 and learning rate of 0.00005 for a maximum of 20 epochs, subject to early stopping. L2-normalization with a strength of 0.01 and dropout of 0.5 was used to regularize models. During MM and UM-WSI training, 2000 patches were sampled from all WSIs belonging to a case each iteration to simplify batching. During sensitivity analysis, 20–10,000 histology patches per case were sampled, and 1–9 axial MRI slices around the tumor center of mass were used. MRI slices were represented as a sequence of patches and concatenated in the patch dimension to maintain symmetry with histology dimensions. Model hyperparameters were selected using a grid search. All models were trained on a single 40GB A100 GPU.

All models were trained to minimize cross-entropy and soft MCC loss and optimized using Adam^45^. Multimodal models are jointly optimized on unimodal and multimodal outputs (Fig. 1). MM-MoE was initially trained for 5 epochs on unimodal inputs without routing to establish a modality-bias for each expert. Subsequent multimodal training was performed for only 15 epochs to maintain parity with other methods.

### Paired data sampling

To randomly sample WSI-MRI pairs from two unimodal datasets, cases are first stratified by label (case_label_table). Since each case consists of only one modality (WSI or MRI), a label-matched case containing the complementary modality is sampled, as described in Algorithm 1. This process is repeated for each case once per epoch. A new random pairing is sampled every epoch.

**Algorithm 1:** Sample paired modalities

**Require:** *case_id*, *label* *case_label_table*

 ⊳ Retrieve histology sample

 **if** CONTAINS(HistoDataset, case_id) **then**

 histo_sample ← GET(HistoDataset, case_id)

 **else**

 eligible_cases ← LOOKUP(case_label_table, (label, “histo”))

 random_id ← RANDOMCHOICE(eligible_cases)

 histo_sample ← GET(HistoDataset, random_id)

 **end** **if**

 ⊳ Retrieve MRI sample

 **if**CONTAINS(MRIDataset, case_id) **then**

 mri_sample ← GET(MRIDataset, case_id)

 **else**

 eligible_cases ← LOOKUP(case_label_table, (label, “mri”))

 random_id ← RANDOMCHOICE(eligible_cases)

 mri_sample ← GET(MRIDataset, random_id)

 **end** **if**

 **return** (histo_sample, mri_sample)

### Metrics

Model performance is primarily reported as accuracy and MCC. MCC is a metric that captures the correlation between predicted and true class labels by incorporating all four elements of the confusion matrix (TP, TN, FP, FN), providing a balanced measure even under class imbalance. AUC refers to the Receiver-Operator Characteristic, i.e., AUROC. Balanced Accuracy (BA) is the macro-average accuracy across all classes. We use the FMI to quantify embedding quality. FMI calculates the geometric mean between two sequences’ pairwise precision and recall, reflecting the proportion of correctly co-clustered pairs relative to all possible pairs.

### Interpretability analyses

Attention values for each patch were computed from the attention pooling layer in the patch-sequence experts. Feature values used for visualization and SHAP analysis were taken from MM-MoE prior to the classification head. SHAP analysis was performed on only the test set using the default Explainer module. Feature SHAP values for each mutation were calculated by grouping labels by mutation status and comparing contribution patterns across the two groups. For each feature, the contribution towards the output logit of the corresponding label was computed in both the positive group (e.g., IDH-mutant cases) and the negative group (e.g., IDH-wild-type cases). The feature vectors are averaged and normalized to yield a feature-importance profile, which can be plotted to show which features most influence the model’s assessment of molecular status.

The five features with the highest SHAP values for a class or mutation were visualized for 16 randomly selected cases and reviewed by a radiologist and neuropathologist. The radiologist was provided with feature maps as seen in Fig. 5f and asked to find trends distinguishing high and low values for each feature. The neuropathologist was given a heatmap of feature distribution on each WSI as well as 10 high and 10 low feature-value patches randomly sampled from the entire dataset.

## Supplementary information

Supplementary Information

## Data Availability

The results shown here are based in whole or in part on data generated by the TCGA Research Network: https://cancergenome.nih.gov/. Additional MRI data was sourced from UCSF-PDGM (https://www.cancerimagingarchive.net/collection/ucsf-pdgm/) and EGD (https://www.healthinformationportal.eu/health-information-sources/erasmus-glioma-database). Histopathology data were acquired from EBRAINS (10.25493/WQ48-ZGX) and TCGA (https://portal.gdc.cancer.gov/).

## Code availability

Code is available at https://github.com/csaueres/radio-path-glioma-subtyping.
