# Supplementary material for: Multimodal fusion of pathology and radiology foundation models for WHO 2021 glioma subtyping
Source: NPJ Precis Oncol. 2026 Mar 12;10:118. doi: 10.1038/s41698-026-01366-5 (PMC12996459; doi:10.1038/s41698-026-01366-5)
Supplement: Supplementary file 1 — Supplementary Information [file 41698_2026_1366_MOESM1_ESM.pdf]

S1 Supplemental Results

	CV				TCGA			
Model	Acc.	AUC	BA	MCC	Acc.	AUC	BA	MCC
MRI	0.88±0.01	0.96±0.01	0.80±0.02	0.74±0.03	0.71±0.04	0.82±0.02	0.63±0.04	0.51±0.06
WSI	0.87±0.04	0.94±0.02	0.84±0.03	0.75±0.07	0.80±0.03	0.91±0.01	0.77±0.03	0.67±0.04
LF	0.90±0.03	0.98±0.01	0.90±0.04	0.81±0.05	0.80±0.05	0.93±0.02	0.80±0.04	0.70±0.07
EF	0.90±0.03	0.97±0.01	0.88±0.03	0.80±0.04	0.83±0.02	0.93±0.01	0.78±0.02	0.71±0.02
MoE	**0.94±0.02**	**0.99±0.00**	**0.91±0.02**	**0.87±0.04**	**0.85±0.03**	**0.94±0.02**	**0.80±0.03**	**0.73±0.05**

Table S1 Extended results for Figure 1. Performance for patch-sequence models across different metrics during cross-validation and on the hold-out TCGA test set. MoE shows the strongest performance across all metrics. LF = Late Fusion, EF = Early Fusion, MoE = Mixture of Experts. Acc. = Accuracy, BA = Balanced Accuracy, MCC = Matthew's Correlation Coefficient. The best result in each column is highlighted in bold. Results given as mean ± standard deviation across 5 folds.

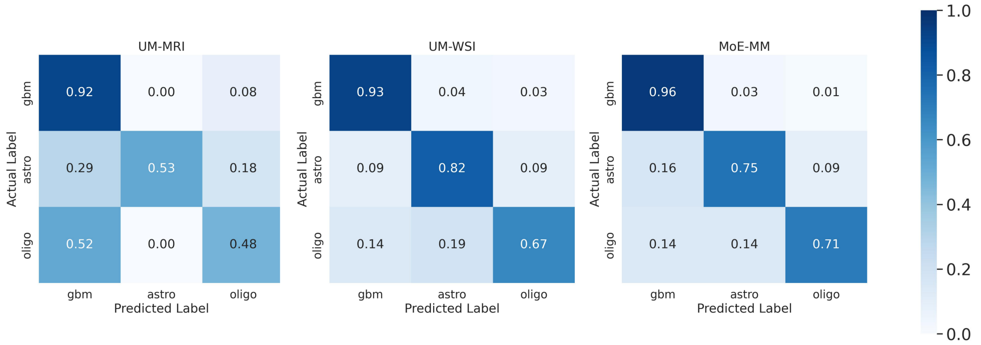

Fig. S1 Model Prediction Confusion Matrices. Generated on TCGA dataset; percentages normalized across rows. UM-MRI (Left): Unimodal MRI model demonstrates strong glioblastoma bias with poor performance on low grade gliomas. UM-WSI (Middle): Unimodal histology model performs similarly to UM-MRI on glioblastomas but has superior low grade glioma discrmination. Misclassified cases are evenly distributed. MoE-MM (Right): Improved glioblastoma performance. Mistakes more astrocytomas for glioblastomas relative to histology model.

In order to better characterize our model's robustness to varying amounts of input data, we performed two experiments varying the number of patches for each modality seen per training sample. For WSI, the number of sampled patches was varied between 20 and 10000, and for MRI up to 8 additional axial slices were fed into the

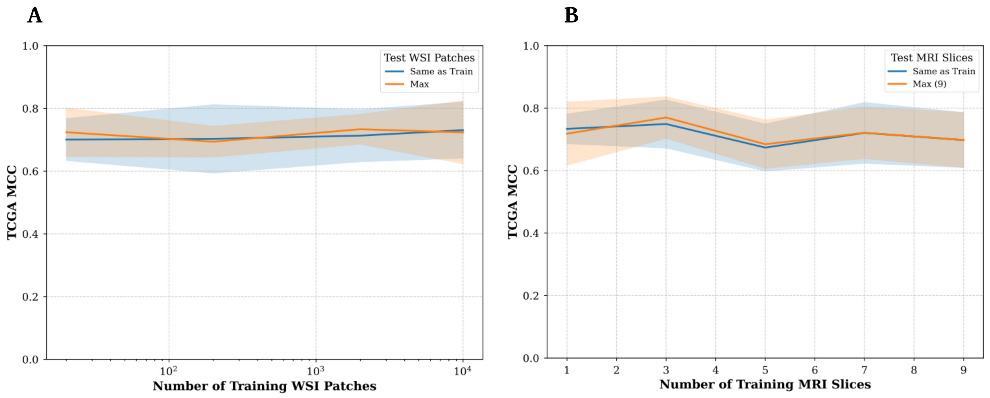

Fig. S2 Sensitivity Analysis of MoE-MM model. A) Test MCC vs. number of sampled histology patches per case. Performance stays constant as the number of patches increases. B) Test MCC vs. number of MRI slices. Performance stays constant as the number of slices increases. This trend is the same when testing on all available slices (orange) or testing on the same number of slices used to train the model (blue).

model. Neither additional MRI or WSI context had a meaningful effect on model performance(Fig. S2). This finding held both when models were evaluated on the same number of patches/slices as during training and on all available slices. The model performed well even at low patch counts, suggesting its ability to be used in conjunction with biopsy data, which offer little tissue. 2000 training histology patches and 1 training MRI slice were found to have the smallest standard deviation, indicating consistent performance across folds.

Institution	Dataset	Count	Accuracy
HFH	GBM	30	0.93 ± 0.04
ACC	GBM	16	0.96 ± 0.04
UCSF	GBM	11	0.87 ± 0.11
CW	GBM	8	0.93 ± 0.09
DU	GBM	6	1.00 ± 0.00
EU	GBM	4	0.98 ± 0.08
INCB	GBM	3	1.00 ± 0.00
HFH	LGG	44	0.78 ± 0.04
CW2	LGG	26	0.77 ± 0.09
TJU	LGG	15	0.70 ± 0.10
CW	LGG	8	0.88 ± 0.10

Table S2 MoE-MM multimodal accuracy stratified by TCGA acquisition site. Institutions contributed to TCGA-GBM collection, TCGA-LGG, or both. Performance within each dataset is consistent across acquisition site, with TCGA-GBM accuracy largely reaching over 90%, and TCGA-LGG between 70-80%. Accuracy reported as mean $\pm$ std over 10 folds.

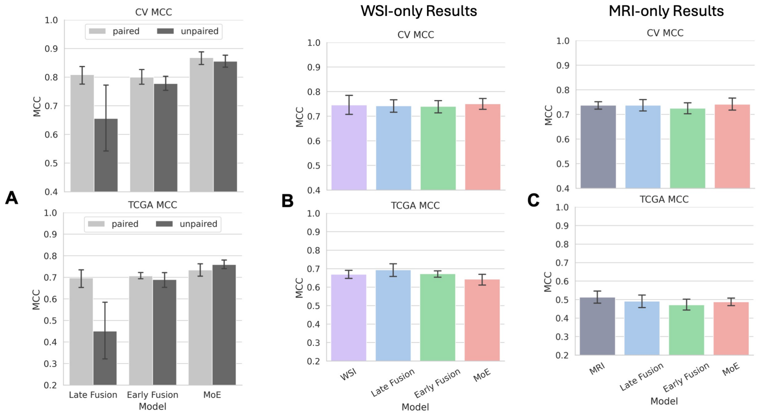

Fig. S3 Extended Version of Figure 3. The trends observed on TCGA also hold for cross validation (CV).

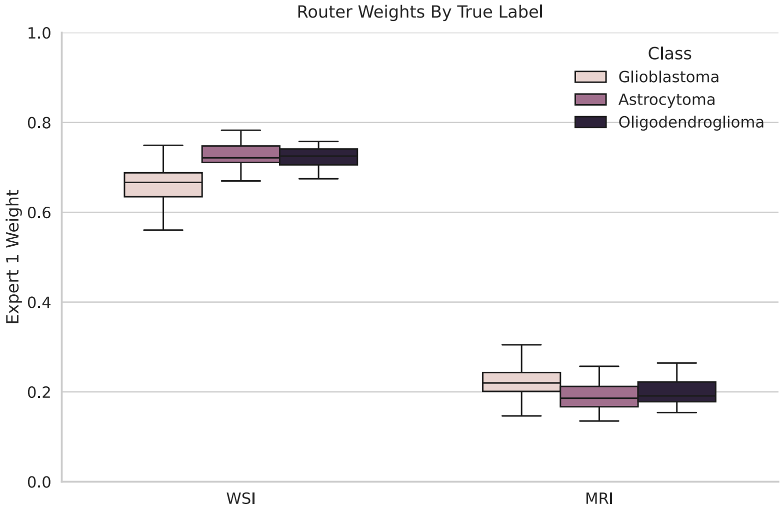

Fig. S4 Boxplot of router weights, grouped by subtype. Computed from MM-MoE model on TCGA dataset. Expert 1 is assigned more weight for WSIs and less for MRIs. Expert 2 (not shown) is weighted as (1-Expert 1 weight), and therefore specializes more in MRIs. The spread is relatively small, indicating that router weights are not as dynamic as expected. Glioblastomas receive the most balanced expert contribution.

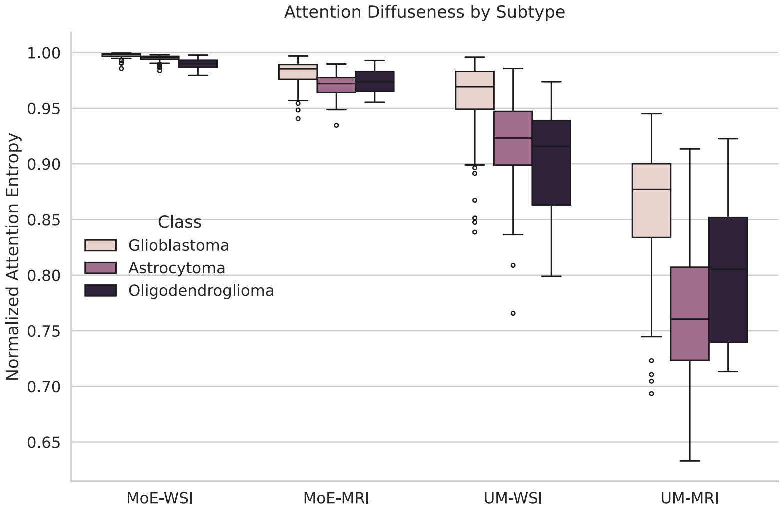

Fig. S5 Boxplot of patch attention entropy, grouped by subtype. Glioblastomas have the highest entropy, equating to most diffuse attention.
